# TNFRSF10B Implicated in Osteoarthritis Protection via the Alpha-Tocopherol-to-Sulfate Ratio: A Multiomics and Mendelian Randomization Study

**DOI:** 10.3390/ijms27188382

**Published:** 2026-09-20

**Authors:** Qichang Gao, Tuo Shao, Yiming Ma, Zhange Yu, Jiaao Gu, Keying Yuan

**Affiliations:** 1 Department of Central Laboratory, First Affiliated Hospital of Harbin Medical University, Harbin 150081, China; 202201199@hrbmu.edu.cn (Q.G.); shaotuo@hrbmu.edu.cn (T.S.); 2 Department of Pharmacology, Harbin Medical University, Harbin 150081, China; 3 Department of Orthopedic Surgery, First Affiliated Hospital of Harbin Medical University, Harbin 150081, China; yimingma@hrbmu.edu.cn (Y.M.); yuzhange1967@163.com (Z.Y.)

**Keywords:** osteoarthritis, TNFRSF10B, Mendelian randomization, machine learning, single-nucleus RNA sequencing, alpha-tocopherol-to-sulfate ratio

## Abstract

Osteoarthritis (OA) is a chronic inflammatory and degenerative joint disease that commonly affects the aging population. This study was designed to decipher gene–metabolite regulatory networks driving OA progression via combined computational prediction and experimental validation. Differential gene expression analysis, weighted gene coexpression network analysis, and machine learning algorithms were integrated to screen for key regulatory genes. To infer causal associations between these candidates and OA susceptibility, we performed Mendelian randomization (MR) using cis-expression quantitative trait loci (cis-eQTL) as genetic instruments and identified significant associations between TNFRSF10B and OA. In parallel, single-nucleus RNA sequencing (snRNA-seq) was used to map the cell-type-specific expression profile of TNFRSF10B, and its expression was validated in human knee synovial tissues. The protective effect of TNFRSF10B on OA was statistically mediated in part by the alpha-tocopherol-to-sulfate ratio, as inferred from Mendelian randomization analysis, with the indirect effect accounting for 16.85% (95% CI: 2.96–30.74%) of the total protective effect. snRNA-seq data revealed that TNFRSF10B was widely expressed across synovial cell populations but reduced in OA samples. Collectively, this study identified TNFRSF10B as a candidate protective factor in OA, supported by genetic evidence from MR analysis and reduced expression in OA synovial tissues, offering statistical evidence for gene–metabolite interplay in OA pathogenesis.

## 1. Introduction

Osteoarthritis (OA) is a chronic inflammatory and degenerative joint disease that is highly prevalent in the aging population and a leading cause of physical disability and chronic pain [1]. Epidemiological data indicate that the global prevalence of OA has increased by 132.2% over the past three decades and is projected to rise by 60–100% by 2050, representing a major public health burden [2]. Although current clinical therapies can alleviate pain and ameliorate symptoms, they cannot reverse joint degeneration. This therapeutic gap arises primarily from an incomplete understanding of the molecular regulatory mechanisms and metabolic pathways underlying OA pathogenesis, which has hindered the development of precise intervention targets.

Accumulating evidence indicates that OA is not a homogeneous disease entity; systemic metabolic imbalance, chronic low-grade inflammation, and genetic variations all contribute to its development and progression [3,4,5]. Owing to their ease of detection, plasma metabolites have emerged as attractive candidates in biomarker research. Rockel et al. conducted a plasma metabolomics analysis on hundreds of patients with knee OA and reported significant alterations in vitamin B9 and B12 metabolism [6]; earlier clinical studies have demonstrated that supplementation with these vitamins can improve symptoms and enhance treatment outcomes [7,8]. Elevated plasma phenylalanine levels have also been associated with radiographic progression in patients with knee OA [9], and a combination of succinate, xanthine, and L-tryptophan has been proposed as an adjunctive diagnostic tool [10]. Thus, plasma metabolites are highly important in the pathogenesis, diagnosis, and treatment of OA.

Multiomics bioinformatic approaches provide powerful tools for identifying core molecular targets in OA [11,12,13]. By integrating machine learning with multiomics data, researchers have identified several promising diagnostic biomarkers for OA. Mendelian randomization (MR) offers unique advantages for establishing causal relationships between genetic variants and disease outcomes, effectively mitigating the confounding and bias inherent in observational bioinformatic analyses [14]. Although MR has been applied in a small number of studies to identify OA-associated biomarkers or cytokines [15,16], the full gene–metabolite–disease regulatory axis remains poorly characterized, and mediation analyses to delineate the underlying metabolic mechanisms remain scarce. In this study, we integrated transcriptomic profiling, multiple machine learning algorithms, and MR to investigate the regulatory roles of hub genes and plasma metabolites in OA progression, followed by validation in clinical specimens. We aimed to elucidate a gene–metabolite regulatory network in OA and provide novel evidence for precision diagnosis and targeted therapy.

## 2. Results

### 2.1. Identification of Differentially Expressed Genes in OA and Functional Enrichment Analysis

**Figure 1:**
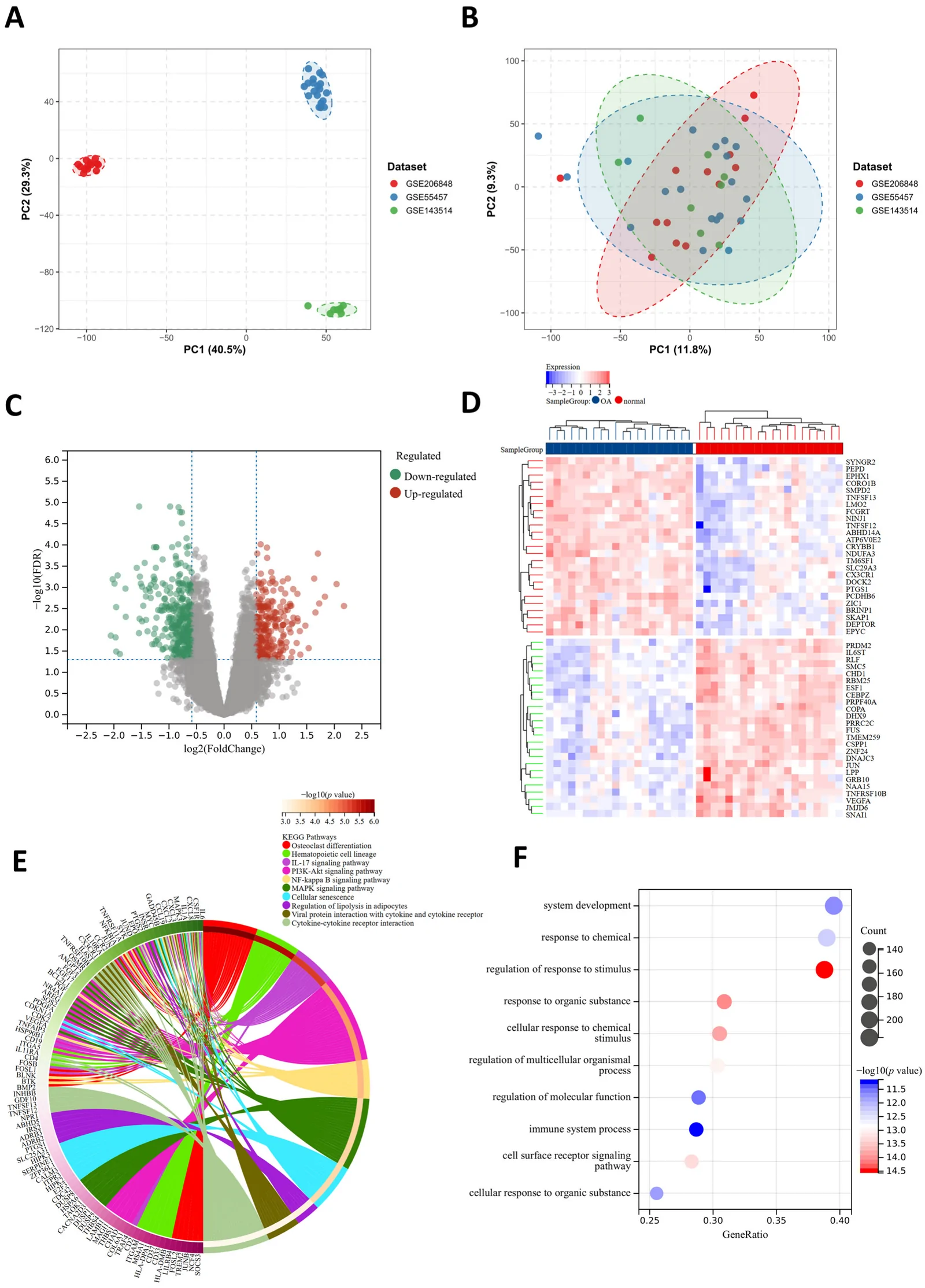
Integrative transcriptomic profiling of OA synovium across three public datasets. (**A**,**B**) PCA plots displaying the sample distribution before (**A**) and after (**B**) batch correction for the merged GSE206848, GSE55457, and GSE143514 cohorts, colored according to the dataset of origin. The axes represent the first two principal components (PC1 and PC2), with the percentage of variance explained indicated in parentheses. (**C**) Volcano plot used to visualize the differential gene expression profiles between OA and healthy synovial samples. (**D**) Heatmap showing the top DEGs with significant differences in expression patterns between diseased and healthy tissues. (**E**) Circular network diagram summarizing the enrichment of DEGs in representative signaling pathways. (**F**) Bubble plot displaying the GO biological process terms with the highest enrichment; the x-axis represents the gene ratio, the bubble size corresponds to the number of enriched genes, and the color represents the −log *p* value.

After the three training datasets (GSE206848, GSE55457, and GSE143514) were merged and batch correction was performed, 40 samples (20 OA patients and 20 healthy controls) were retained for analysis (Figure 1A,B). Differential expression analysis revealed 617 significantly dysregulated genes in OA synovial tissue compared with normal tissue, including 247 upregulated and 370 downregulated genes (Figure 1C,D). The analysis results indicated that pathways, including the IL-17 signaling pathway and other inflammation-related pathways, as well as pathways regulating osteoclast differentiation and cellular senescence, were significantly enriched. These pathways are closely related to the core pathological processes of osteoarthritis (Figure 1E). Additionally, enrichment of immune system processes and regulation of the response to stimuli were observed, reflecting key OA pathologies such as immune-inflammatory activation, cellular stress responses, and aberrant signal transduction. For the cellular component category, enrichment was detected in the extracellular region, vesicular structures, and plasma membrane components, indicating broad involvement of the cell membrane and extracellular microenvironment in intercellular communication and local tissue remodeling. For molecular functions, enrichment was dominated by core sequence-specific transcriptional regulatory activity (Figure 1F, Supplementary Figure S1A,B).

### 2.2. Identification of Key Modules via WGCNA

**Figure 2:**
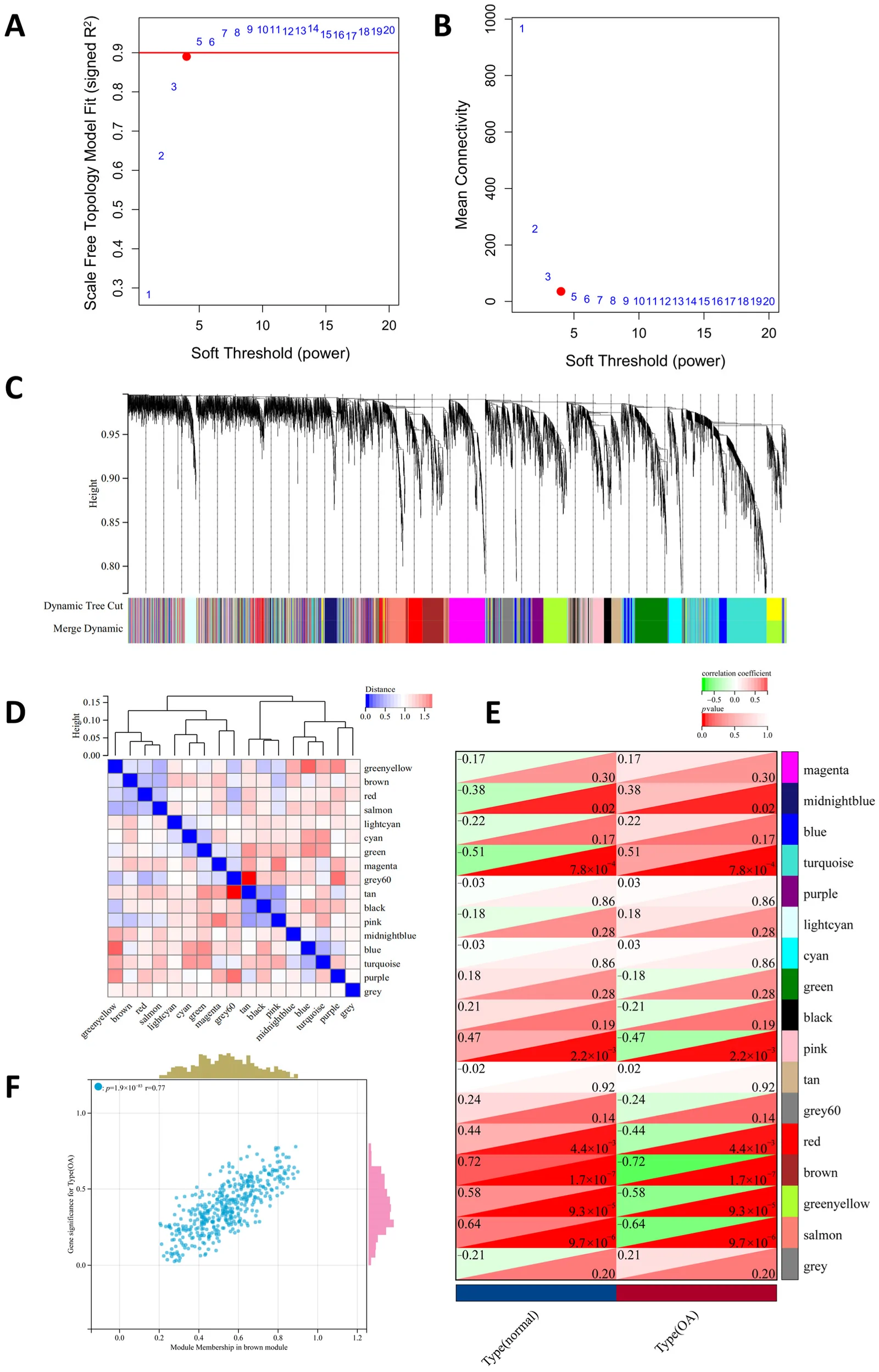
WGCNA identified OA-associated gene modules in the integrated expression cohort. (**A**) Determination of the optimal soft threshold power based on the scale-free topology model fit (signed R^2^). (**B**) Mean connectivity within the range of candidate power values. (**C**) Gene clustering tree diagram constructed on the basis of the difference in topological overlap. The colored strips below the tree diagram represent the module allocation obtained through dynamic tree pruning, as well as the final merged module set. (**D**) Heatmap showing the distances between module eigengenes, demonstrating the independence between modules. (**E**) Heatmap of the associations between modules and phenotypes; each entry shows the Pearson correlation coefficient and corresponding *p* value between the principal component of a specific module and the OA disease phenotype. (**F**) Scatter plot demonstrating the correlation between the significance score of OA genes and the membership index of genes within the brown co-expressed module, verifying the robust correspondence between the core gene state within the module and the disease correlation.

**Figure 3:**
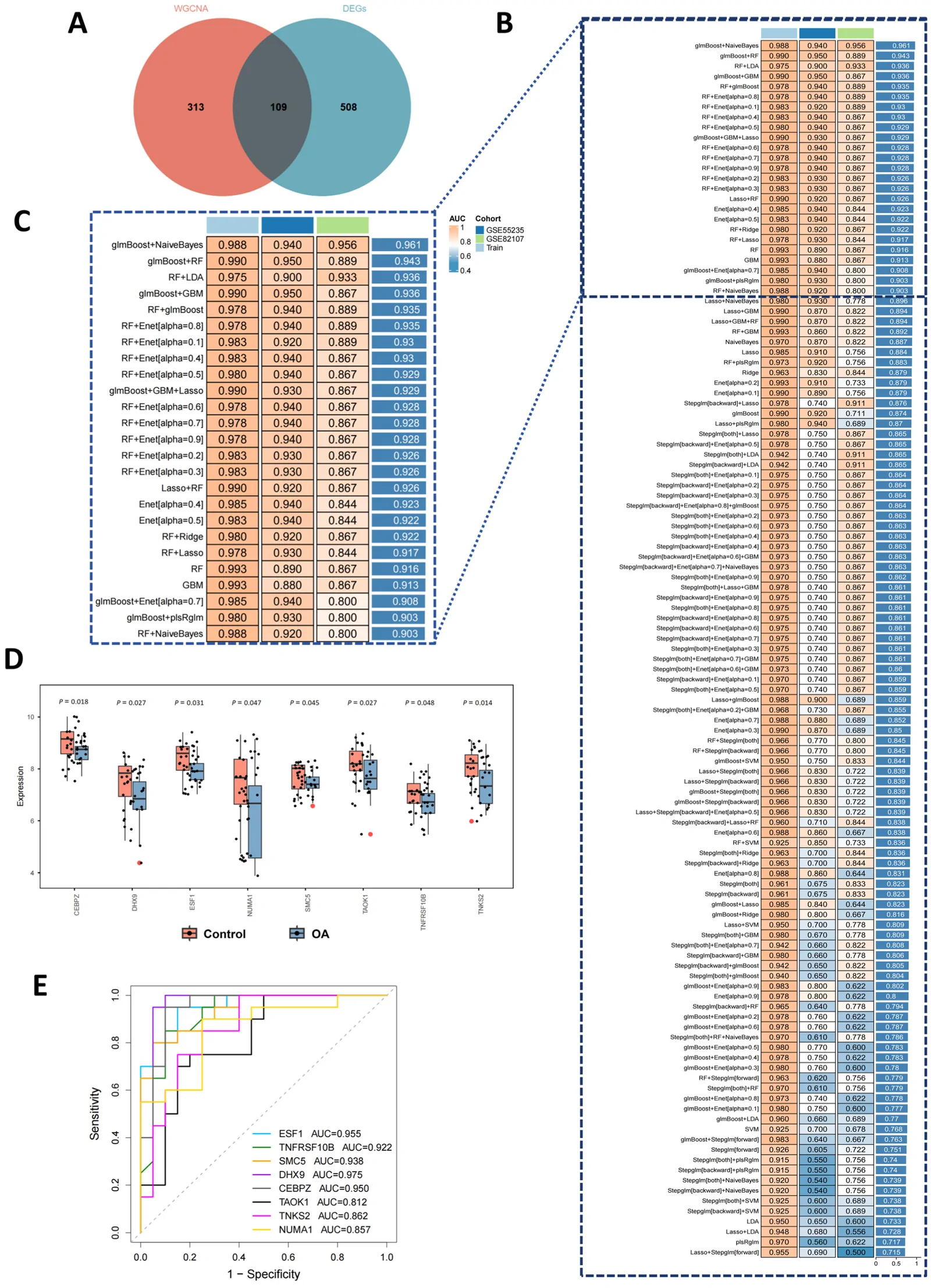
Machine learning-based identification and validation of diagnostic gene biomarkers for OA. (**A**) Venn diagram showing the intersection of the genes in the WGCNA modules related to OA and the list of differentially expressed genes, which defines the core candidate genome for the subsequent construction of the diagnostic model. (**B**) Heatmap showing the AUC values obtained by 119 machine learning model variants in the training set and two external validation datasets (GSE55235 and GSE82107). (**C**) Detailed view of the high-scoring models in the training and validation datasets with an average AUC > 0.9. (**D**) Box plot comparing the expression levels of eight core diagnostic genes in OA samples and control samples. (**E**) ROC curve for each diagnostic gene, where the AUC value reflects its individual discriminative ability for OA.

The optimal soft-threshold power (β = 4) was determined on the basis of a scale-free topology fit index (R^2^) of 0.86 and preserved network connectivity, which is consistent with the properties of biological networks (Figure 2A,B). Gene expression profiles were then used to calculate the topological overlap matrix (TOM), followed by hierarchical clustering to identify 17 distinct coexpression modules (Figure 2C). Intermodule correlation analysis confirmed the independence of these modules (Figure 2D). Correlation analysis between MEs and OA status revealed that the brown module had the strongest negative association (r = −0.72, *p* = 1.7 × 10^−7^) (Figure 2E). Within this module, a robust positive relationship between module membership and gene significance further supported its biological relevance as a candidate pool for OA regulatory genes (Figure 2F). To enhance biological reliability, genes from the brown module were intersected with the previously identified DEGs, yielding 109 candidate core genes (Figure 3A). Using the cytoHubba plugin in Cytoscape (v3.10.0), the top 50 hub genes were retained for subsequent machine learning-based diagnostic model construction and validation (Supplementary Figure S1C, Supplementary Table S3).

### 2.3. Machine Learning-Based Identification of Diagnostic Gene Biomarkers for OA

Among the models tested, 25 combinations, including glmBoost + NaiveBayes, achieved mean area under the curve (AUC) values above 0.9 across all datasets (Figure 3B,C). An eight-gene panel comprising *ESF1*, *TNFRSF10B*, *SMC5*, *DHX9*, *CEBPZ*, *TAOK1*, *TNKS2*, and *NUMA1* was ultimately selected as the core diagnostic gene signature for OA. The transcription profiles of these genes are shown in Figure 3D. The diagnostic potential of each individual gene was assessed via receiver operating characteristic (ROC) curve analysis; all eight genes exhibited strong discriminatory power (AUC ranging from 0.812 to 0.975) (Figure 3E), supporting their potential clinical utility as diagnostic biomarkers.

### 2.4. Molecular Subtypes of OA and Characterization of the Immune Microenvironment

**Figure 4:**
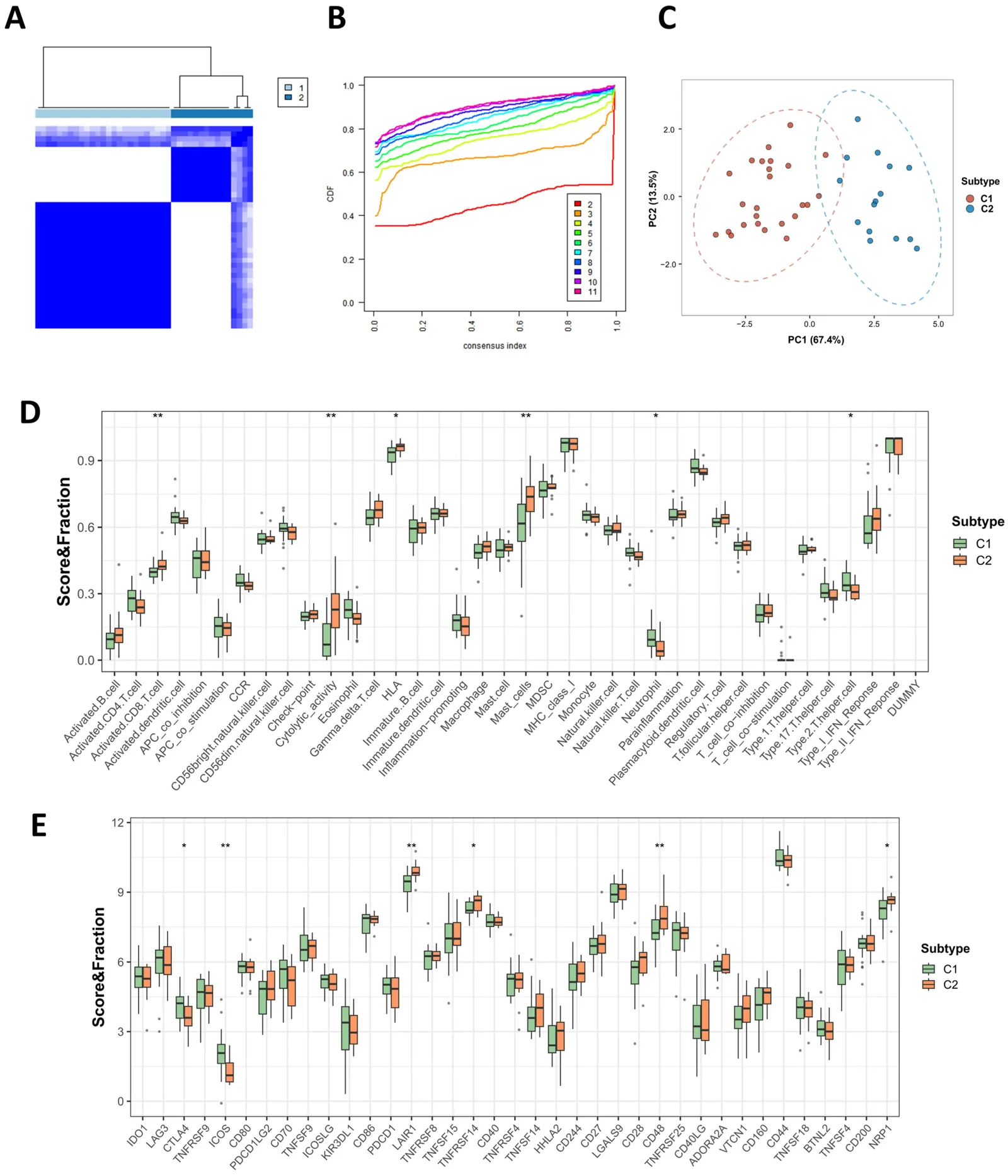
Unsupervised consensus clustering defines two OA molecular subtypes with distinct immune microenvironments. (**A**) The consensus clustering matrix when k = 2 shows that the OA samples are stably divided into two molecular subtypes (C1 and C2). (**B**) The CDF curve of the consensus index from 2–10 was used to determine the optimal clustering scheme. (**C**) Principal component analysis graph showing a significant separation between the C1 and C2 subtypes at the transcriptomic level. (**D**) Comparison of the abundance of infiltrating immune cells and the activity scores of immune pathways between the two subtypes. (**E**) Differential expression of immune checkpoint molecules in the C1 and C2 subtypes. * *p* < 0.05, ** *p* < 0.01.

Unsupervised clustering analysis was used to stratify OA samples into molecular subtypes. By determining the optimal number of clusters, k = 2, two stable expression profile subtypes, C1 and C2, were obtained (Figure 4A–C). Compared with the C1 subtype, the C2 subtype exhibited significantly higher levels of activated CD8^+^ T cells, cytolytic activity, and mast cell infiltration (*p* < 0.01), suggesting its immunostimulatory phenotype and significant differences in the local joint immune microenvironment (Figure 4D). Further analysis of the differences in immune checkpoint molecules revealed that *ICOS*, *LAIR1*, and *CD48* were significantly dysregulated in the C1 subtype (*p* < 0.01) (Figure 4E).

### 2.5. MR Analysis Provides Statistical Evidence for a Causal Association Between TNFRSF10B and OA

**Figure 5:**
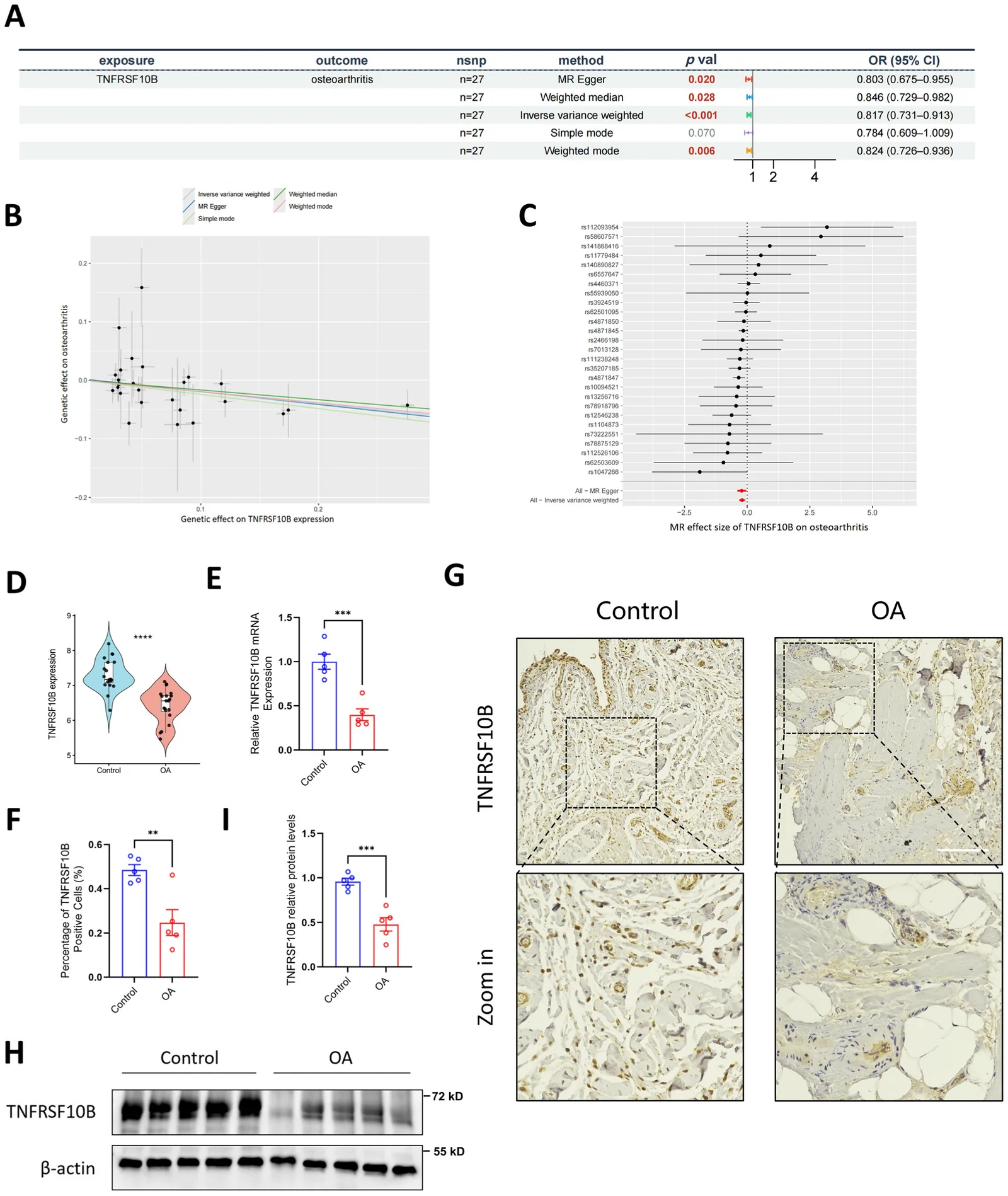
MR analysis and clinical validation established TNFRSF10B as a protective factor in OA. (**A**) Summary table and forest plot of MR estimates for the effect of gene-predicted TNFRSF10B expression on the risk of OA. (**B**) Scatter plot of each single-nucleotide polymorphism (SNP) on the relationship between TNFRSF10B expression and the risk of OA; the fitted straight line represents the causal estimates obtained by each MR method. (**C**) Forest plot of individual SNP-level causal effects of TNFRSF10B in OA. (**D**) Violin plot of TNFRSF10B transcript levels in OA and normal synovium in the integrated transcriptome cohort. (**E**) RT-qPCR verification of the decreased TNFRSF10B mRNA expression level in clinical OA synovial samples. (**F**) Quantitative immunohistochemical analysis of TNFRSF10B-positive cells revealed a significant reduction in the proportion of this protein in OA tissues. (**G**) Representative immunohistochemical staining of the TNFRSF10B protein in normal and OA synovium. Scale bars: 200 μm; the inset shows an enlarged view of the boxed area. (**H**) Representative Western blot images of the TNFRSF10B protein in OA patient and healthy control synovial tissues. (**I**) Densitometric analysis of TNFRSF10B levels, with β-actin used as a reference. ** *p* < 0.01, *** *p* < 0.001, **** *p* < 0.0001.

Using the core diagnostic genes selected in the previous analysis, we systematically evaluated the causal relationship between the predicted gene expression levels and susceptibility to OA. Among all the candidate genes, only TNFRSF10B had a statistically significant causal relationship with the risk of OA (*p* < 0.05). The upregulation of TNFRSF10B at the gene level was associated with a reduced risk of OA, suggesting a potential protective effect during the disease process. A consistent direction of effect across alternative MR models reinforced the primary finding (Figure 5A–C). The Cochran’s Q test did not detect significant heterogeneity among the instruments, and the MR–Egger intercept test also did not reveal directional pleiotropy (see detailed statistical information in Supplementary Table S4). To assess the cross-cohort stability of this association, we performed phenotypic sensitivity analyses using two additional osteoarthritis GWAS datasets from the same study with distinct phenotype definitions: ebi-a-GCST005813 and ebi-a-GCST005814. In both replication cohorts, IVW analysis consistently reproduced the significant protective effect of increased TNFRSF10B expression (GCST005813: OR = 0.792, *p* = 0.001; GCST005814: OR = 0.910, *p* = 0.037). The direction of the effect was fully consistent across all the complementary MR methods, although not all reached statistical significance. No significant heterogeneity or directional pleiotropy was observed in sensitivity tests across the three datasets (Supplementary Tables S4 and S5).

To further substantiate the biological plausibility of this causal inference, we validated TNFRSF10B expression profiles in OA synovial tissues at both the transcriptional and translational levels. Analysis of the aforementioned integrated transcriptomic cohort revealed that TNFRSF10B transcript abundance was significantly lower in OA synovium samples than in healthy control samples (*p* < 0.0001; Figure 5D). Consistently, RT-qPCR analysis of clinical samples revealed a marked downregulation of TNFRSF10B mRNA in the OA group (*p* < 0.001) (Figure 5E). Immunohistochemical staining further verified that the proportion of TNFRSF10B-positive cells was significantly lower in OA synovial tissues than in normal control tissues (*p* < 0.01) (Figure 5F,G). Consistent with these findings, Western blot analysis revealed significantly lower TNFRSF10B protein levels in OA synovial tissues than in control tissues after normalization to β-actin (Figure 5H,I).

### 2.6. snRNA-Seq Reveals Dysregulation of TNFRSF10B in OA

**Figure 6:**
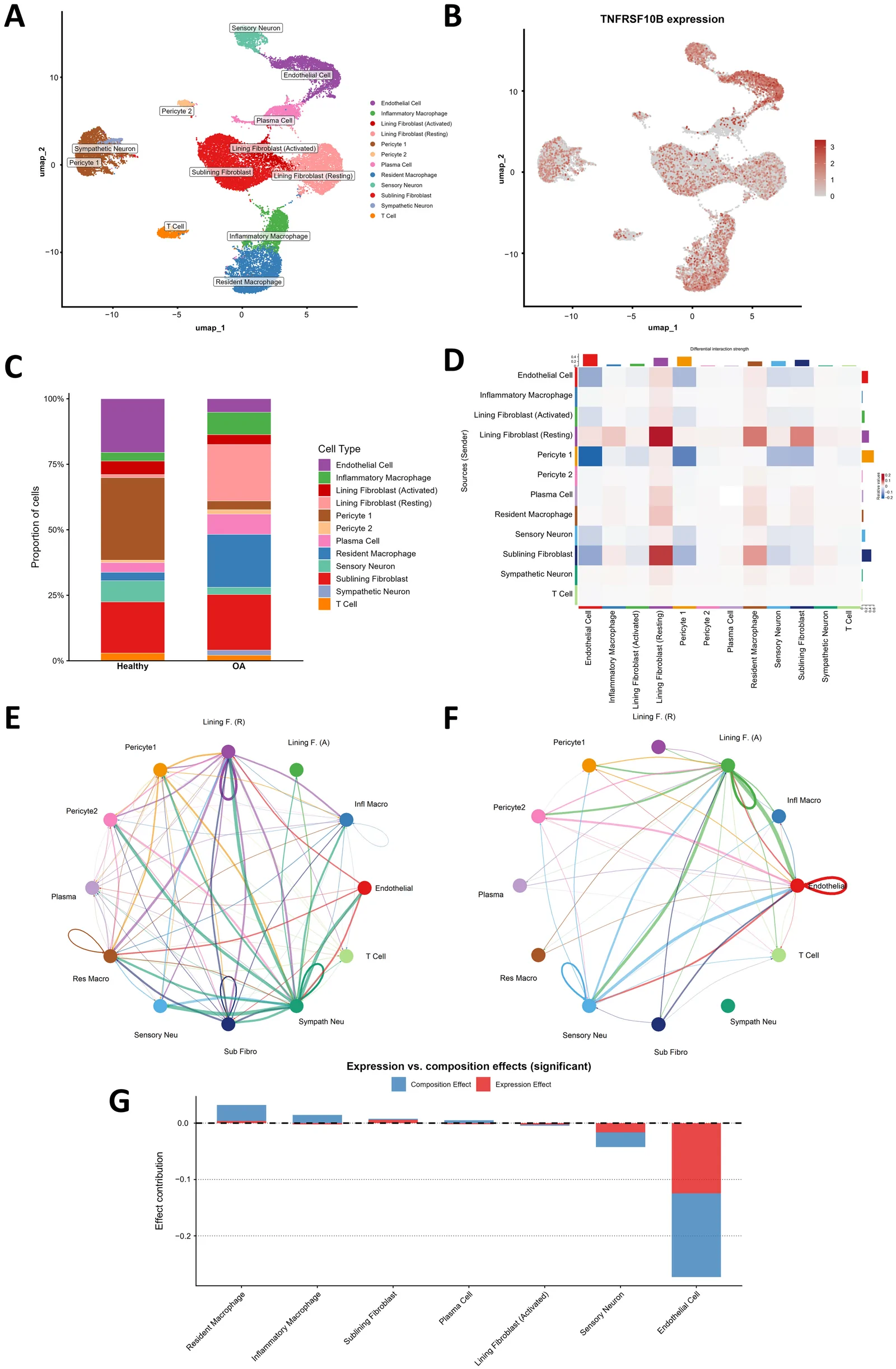
Synovial cell atlas, cell–cell communication reprogramming, and quantitative decomposition of TNFRSF10B alterations in OA. (**A**) UMAP visualization map of 12 different cell populations identified from the synovial snRNA-seq data, colored according to the annotated cell types. (**B**) UMAP map showing the expression distribution of TNFRSF10B in all cell nuclei, with the color gradient representing the log-normalized expression level. (**C**) The stacked bar chart shows the relative proportions of each cell population in healthy and osteoarthritis synovial tissues. (**D**) The heatmap displays the pairwise differences in ligand-receptor interaction strength between different cell types, comparing OA and healthy synovial tissues. Red indicates enhanced interaction in OA; blue indicates weakened interaction in OA. (**E**,**F**) Arc diagrams showing the global communication network between cells in healthy and OA synovial tissues. The size of the nodes reflects the total interaction degree of each cell type, and the thickness of the edges corresponds to the inferred interaction strength. (**G**) Stacked bar plot decomposing the total tissue-level change in TNFRSF10B in OA into an expression effect (mean expression change weighted by healthy cell proportion) and a composition effect (cell proportion change weighted by healthy mean expression). Only cell types with significant differential expression (BH-adjusted *p* < 0.05) are shown. Positive, net increase; negative, net decrease.

To characterize the cellular expression landscape of TNFRSF10B in OA, we analyzed a publicly available single-nucleus RNA-seq dataset (GSE216651) of human synovial tissues [17]. Unsupervised clustering and UMAP visualization identified 12 transcriptionally distinct cell populations (Figure 6A, Supplementary Figure S2A), including Sublining Fibroblast, Lining Fibroblast (Resting), Lining Fibroblast (Activated), Endothelial Cell, Resident Macrophage, Inflammatory Macrophage, Sensory Neuron, Sympathetic Neuron, Pericyte 1, Pericyte 2, Plasma Cell, and T-Cell populations. TNFRSF10B was widely expressed in various cell types (Figure 6B), but it was overall downregulated in OA patients compared with healthy controls (Supplementary Figure S2B–D), confirming our previous research results. The OA synovium also showed significant compositional remodeling: lining fibroblasts (resting) and resident macrophages constitute the main cell populations, whereas the proportions of endothelial cells and pericytes are significantly reduced (Figure 6C).

The intercellular communication network has also undergone directional reprogramming. Sympathetic neurons became the strongest signal sender, acting mainly on sensory neurons, fibroblasts, and endothelial cells, whereas the signals received by endothelial cells are generally weakened, including those from lining fibroblasts (Activated), sensory neurons, and pericytes 2 (Figure 6D–F). To dissect the contributions of changes in intracellular expression and the cell proportion to the overall change in TNFRSF10B at the tissue level, we performed effect decomposition analysis (Figure 6G). Endothelial cells made the most significant net negative contribution, indicating that reduced TNFRSF10B expression per endothelial cell and concurrent loss of endothelial cells jointly drove the overall downregulation of TNFRSF10B in the OA synovium. In contrast, the positive effects shown by Resident Macrophages were primarily driven by alterations in cell composition (relative proportion) rather than intracellular expression changes. The specific mechanisms driving this compositional shift remain to be elucidated. In summary, these observations generate the hypothesis that loss of TNFRSF10B may be associated with altered intercellular communication in the OA synovium, particularly involving endothelial cells, sympathetic neurons, and fibroblast-macrophage interactions. Future functional studies are needed to test this hypothesis.

### 2.7. MR Analysis of the TNFRSF10B–Metabolite–OA Regulatory Axis

**Figure 7:**
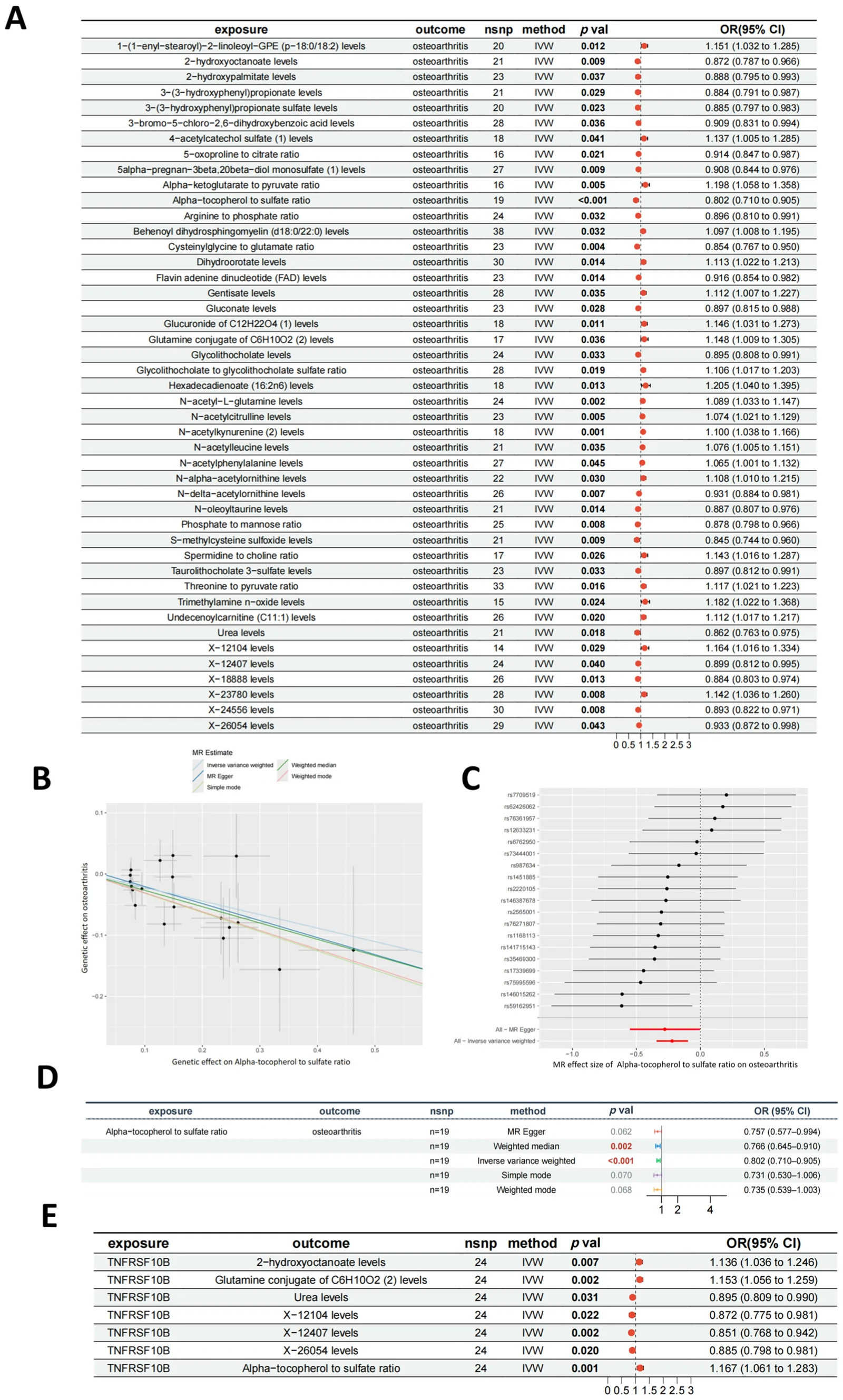
MR delineates the TNFRSF10B–metabolite–OA causal regulatory axis. (**A**) Summary of MR findings identifying metabolites with significant causal associations with OA risk. (**B**) Scatter plot of SNP-level genetic effects on the alpha-tocopherol-to-sulfate ratio versus OA risk, with fitted lines showing causal estimates from complementary MR methods. (**C**) Forest plot of individual SNP causal effect estimates for the alpha-tocopherol-to-sulfate ratio on OA. (**D**) Summary table and forest plot of MR estimates for the effect of the alpha-tocopherol-to-sulfate ratio on OA risk. (**E**) MR estimates for the effect of genetically predicted TNFRSF10B expression on candidate mediator metabolites.

We applied a two-step mediation MR framework to systematically trace the causal chain from TNFRSF10B through plasma metabolites to OA. After screening, a total of 45 metabolites were significantly correlated with the risk of OA (Figure 7A–D). We subsequently used these metabolites as outcomes and evaluated them via MR analysis with TNFRSF10B as the exposure factor. The results revealed that 7 metabolites were associated with TNFRSF10B (Figure 7E).

**Figure 8:**
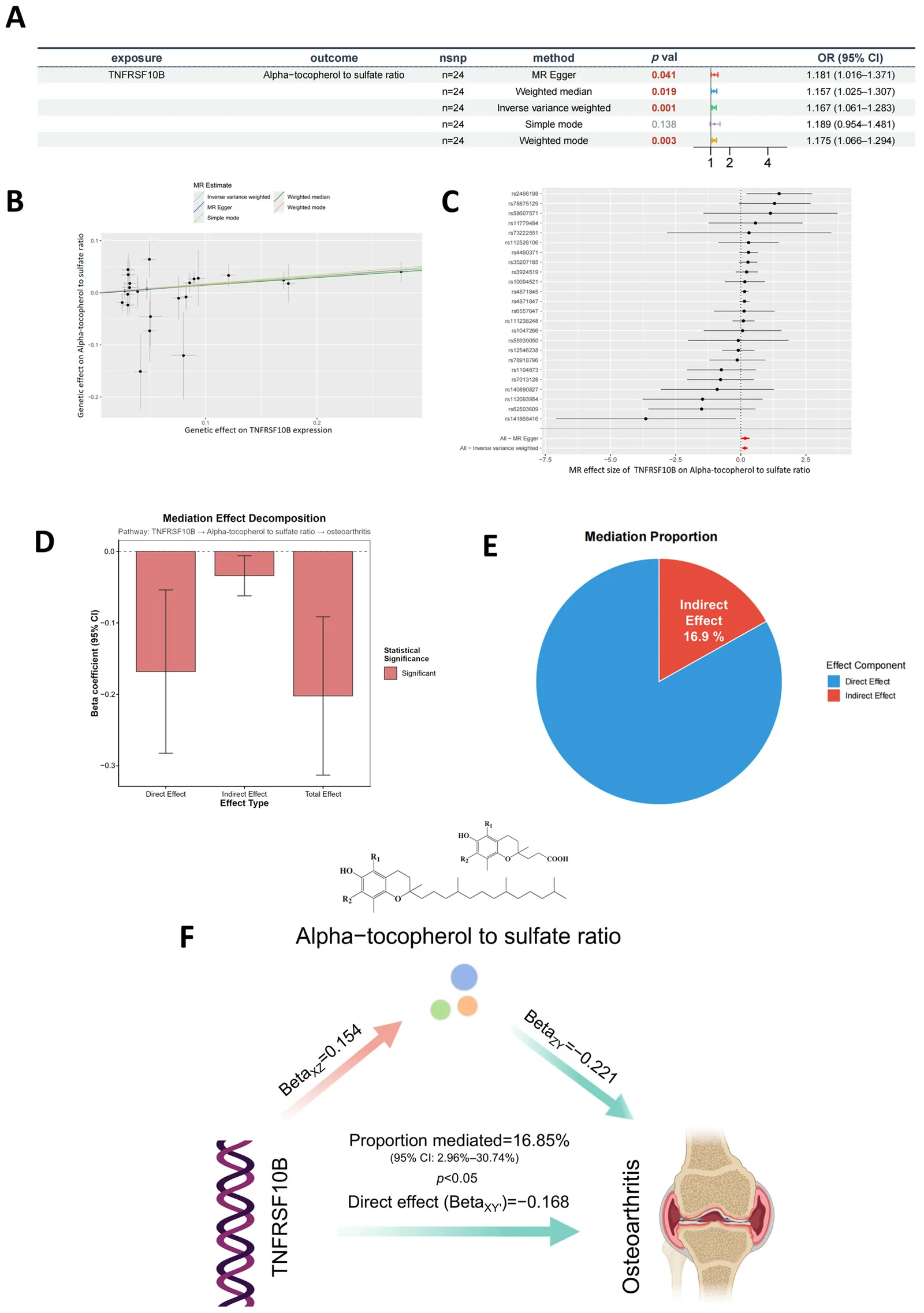
MR analysis of the protective effect of TNFRSF10B on OA through the alpha-tocopherol-to-sulfate ratio. (**A**) Summary table and forest plot of MR estimates for the effect of genetically predicted TNFRSF10B expression on the alpha-tocopherol-to-sulfate ratio. ORs are derived from exponentiated β coefficients for continuous outcomes. (**B**) Scatter plot of SNP-level genetic effects on TNFRSF10B expression against the alpha-tocopherol-to-sulfate ratio; fitted slopes indicate causal estimates from complementary MR methods. (**C**) Forest plot of individual SNP causal effect estimates for TNFRSF10B on the mediator. (**D**) Effect decomposition plot presenting regression coefficients (β) along with their 95% confidence intervals for the direct, indirect (mediated), and total effects of TNFRSF10B on OA risk. (**E**) Pie chart showing the proportion of the total effect indirectly mediated. (**F**) Schematic diagram of the TNFRSF10B–alpha-tocopherol-to-sulfate ratio–OA regulatory axis, annotated with effect estimates.

Among these factors, only the alpha-tocopherol-to-sulfate ratio met the causal significance threshold in both steps and had a statistically significant indirect effect; thus, it represents a candidate mediator between TNFRSF10B and OA (Figure 8A–C, Supplementary Figure S3A–F). Effect decomposition revealed that the indirect effect mediated by the alpha-tocopherol-to-sulfate ratio accounted for 16.85% (95% CI: 2.96–30.74%) of the total protective effect of TNFRSF10B on OA (Figure 8D,E). Complete mediation analysis results, including direct, indirect, and total effects with standard errors, are provided in Supplementary Table S6. These findings indicate that the protective effect of TNFRSF10B on OA is not only achieved through direct pathways but also partially through regulating metabolic homeostasis related to vitamin E, providing a new genetic perspective for understanding the pathogenesis of OA (Figure 8F).

## 3. Discussion

OA is characterized by genetic susceptibility, metabolic disorders, and chronic inflammatory states. Clarifying the integration mechanisms of genes and metabolic regulatory networks provides promising directions for therapeutic development [18,19]. Wang et al. discovered that ARG1 can regulate changes in metabolites derived from the gut microbiota, thereby influencing the progression of OA, and provided a new target for microbiome-based intervention [20]. Multiple gene panels have been widely studied as diagnostic models for OA [21,22,23]; for example, Huang et al. identified a gene spectrum consisting of *CEBPB*, *PTEN*, *ARPC1B*, *PIK3R1*, and *CDC42*, which strongly participates in the aging-driven process of OA [12]. Other studies have constructed diagnostic models of mitochondrial-related genes associated with mitochondrial heterogeneity, providing new strategies for personalized OA treatment [24]. In the present study, we used cis-eQTL genetic instruments and sequentially performed causal MR analysis, phenotypic sensitivity validation, and mediation MR analysis. This approach not only identified TNFRSF10B as a candidate protective gene in OA but also identified the alpha-tocopherol-to-sulfate ratio as a potential mediating variable and quantified its indirect effect as 16.85% of the total effect (*p* < 0.05). From a genetic causal standpoint, these findings provide statistical evidence that this gene is associated with OA pathogenesis via a putative metabolite-mediated pathway. We clarify that MR provides genetic statistical evidence supporting a causal relationship, but the biological mechanism by which TNFRSF10B may influence the alpha-tocopherol-to-sulfate ratio requires experimental validation.

In this study, multiple bioinformatics strategies were combined for TNFRSF10B. WGCNA combined with exhaustive screening across more than 100 machine learning algorithm combinations identified TNFRSF10B as a candidate diagnostic biomarker for OA, a finding supported by multilevel validation in clinical synovial samples. Previous mechanistic work has shown that TNFRSF10B participates in DNA damage responses and apoptosis [25,26] and has been implicated in a range of malignancies and tumor drug resistance [27,28]. Functionally, TNFRSF10B is involved in chemotherapeutic resistance across multiple malignancies [26,27]. At the molecular level, TNFRSF10B functions as a death receptor for TNF-related apoptosis-inducing ligands, mediating a well-characterized apoptotic cascade [29]. An early in vitro study revealed that rheumatoid arthritis synovial fibroblasts express functionally active TNFRSF10B and that an anti-TNFRSF10B monoclonal antibody can trigger the apoptosis of hyperproliferative synovial fibroblasts, thereby preventing synovial hyperplasia, subchondral bone erosion, and cartilage degradation [30]. Studies in a murine antibody-induced arthritis (AIA) model further indicated that the sFasL-TNFRSF10B interaction promotes inflammation through the CX3CL1–CX3CR1 axis [31]. Conversely, other evidence indicates that Tnfrsf10b-knockout mice develop chronic inflammation and are predisposed to tumorigenesis [31,32]. The biological role of TNFRSF10B in degenerative OA remains poorly understood. Although both rheumatoid arthritis and OA affect joints, their core pathogenic mechanisms differ substantially. Using large-scale population genetic data, we established a causal association between elevated TNFRSF10B expression and reduced OA risk. Tissue-level validation revealed that TNFRSF10B mRNA and protein levels were significantly downregulated in the OA synovium, in sharp contrast to the upregulation observed in rheumatoid arthritis, suggesting that TNFRSF10B may perform opposing regulatory functions in different joint diseases. These divergent findings indicate that TNFRSF10B may influence OA progression through a complex signaling network, which warrants further systematic investigation.

Although the presence of sensory and sympathetic neurons in synovial tissue is increasingly recognized, their identification in single-nucleus RNA-seq data from joint tissues is relatively uncommon. In our dataset, we captured these rare populations and therefore performed data-driven annotation based on their top-ranked cluster-specific signature genes. We found that the proportion of sympathetic neurons was significantly increased in the OA synovium and that overall intercellular communication mediated by these neurons was also increased. This change extended to multiple target cell populations, including fibroblasts and immune cells, suggesting the abnormal activation of the sympathetic nervous system in the OA synovial microenvironment. Notably, this enhanced neuronal communication may be putatively linked to TNFRSF10B downregulation, representing a data-driven hypothesis that requires experimental validation. Previous studies have shown that TNFRSF10B promotes the secretion of exosomes containing α-synuclein by microglia, thereby triggering neuroinflammation and neuronal damage [33]. The overall reduction in TNFRSF10B in OA may therefore be associated with enhanced sympathetic signaling, which can further promote fibroblast activation, macrophage polarization, and inflammatory mediator release, driving OA-associated inflammation and pain sensitization. Fibroblast phenotypic heterogeneity and macrophage infiltration and activation are core drivers of synovial fibrosis and chronic inflammation in OA. We observed a marked phenotypic shift in lining fibroblasts in OA, characterized by a reduced proportion of the activated subpopulation and substantial expansion of the resting subpopulation, alongside directional reprogramming of their communicative functions. TNFRSF10B showed opposite expression trends in the two fibroblast subtypes, suggesting a potential role in fibroblast phenotypic regulation.

Among the 1400 circulating metabolites screened in this study, the α-tocopherol-to-sulfate ratio was identified as the primary mediating factor, accounting for 16.85% of the overall protective effect of TNFRSF10B against OA. This ratio reflects the balance between the major active form of vitamin E-unoxidized alpha-tocopherol (α-TOH)–and its water-soluble sulfate metabolites generated via oxidation and thus can concurrently reflect oxidative stress levels and the biological activity of α-TOH [34,35,36]. α-TOH is an indispensable micronutrient with antioxidant, anti-inflammatory, and even anticancer effects, and its metabolites also exhibit functions similar to those of hormones [37,38]. For example, Luo et al. reported that an increase in oxidized α-TOH metabolites in the urine of healthy middle-aged adults was associated with a reduction in insulin resistance [39]. Multiple clinical observational studies have confirmed that vitamin E status is associated with OA. In case–control studies in different countries and regions, low levels of α-tocopherol have consistently been associated with more severe OA [40,41,42]. Although numerous trials have explored the role of vitamin E in disease prevention or treatment, the results are still inconsistent. Some evidence indicates that vitamin E attenuates cartilage matrix degradation by reducing lipid peroxidation [43] and can ameliorate H_2_O_2_-induced chondrocyte senescence and apoptosis [44]. In contrast, vitamin E cannot protect synovial cells from superoxide anion-induced apoptosis [45]. Furthermore, reports have indicated that high-dose vitamin E can cause damage to the skeletal system, potentially via interference with vitamin K metabolism and excretion [46]. This study provides statistical evidence supporting a candidate protective role of vitamin E metabolism in OA and identifies the biological activity of α-TOH and its metabolic regulation as potential targets for precise antioxidant intervention in OA. Overall, our findings provide statistical evidence for a potential metabolic pathway linking TNFRSF10B to OA; however, the molecular mechanisms underlying this association remain to be experimentally validated.

Several limitations should be considered when our findings are interpreted. First and foremost, our MR analysis relied on European-descent GWAS data and blood-derived cis-eQTLs, which may not fully capture synovial-specific expression. This is compounded by the fact that the OA GWAS included mixed hospital-diagnosed cases. As a result, our estimates could be influenced by tissue-specific effects and phenotypic heterogeneity, and extrapolating them to non-European populations or specific OA subtypes needs to be done cautiously. Moving to the computational side, our training cohort comprised only 40 synovial samples. Batch correction was performed to mitigate platform-specific technical variation, but the small sample size may limit the statistical power and generalizability of our findings. While 119 model configurations and two external validation cohorts were used to increase robustness, the small sample size still makes overfitting a legitimate concern. Both the C1/C2 subtype comparison and the MR screening of 1400 plasma metabolites were exploratory without full multiple testing correction, which may increase the risk of false positives; replication in larger, independent cohorts is warranted. We must also acknowledge that the mediation analysis is purely statistical, and the actual mechanism through which TNFRSF10B modulates the metabolic ratio still requires cell and animal experiments. Another caveat is that our control tissues came from trauma patients rather than healthy volunteers, where subtle inflammatory responses may already be present. Thus, the actual mechanism through which TNFRSF10B modulates the metabolic ratio still needs cell and animal experiments. Finally, given the small number of neuronal nuclei captured in the snRNA-seq data, the alterations in neuronal communication should be viewed as hypothesis-generating. Future functional studies are essential to validate these specific observations.

## 4. Materials and Methods

### 4.1. Collection of Human Knee Synovial Samples

Synovial tissue samples were obtained from patients undergoing joint surgery at the Department of Orthopedic Surgery, The First Affiliated Hospital of Harbin Medical University. The OA group consisted of patients with clinically confirmed moderate-to-advanced knee OA who were scheduled for total knee arthroplasty. The exclusion criteria included coexisting rheumatoid arthritis, gouty arthritis, septic arthritis, autoimmune disease, malignancy, and pregnancy or lactation. Healthy control synovial tissue was collected from patients without degenerative joint disease or a history of inflammatory arthritis who underwent knee surgery for traumatic injuries. Detailed demographic and clinical characteristics, including age, sex, and Kellgren-Lawrence grade for each individual, are provided in Supplementary Table S1.

### 4.2. Public Dataset Sources

The transcriptomic data analysed in this study were obtained from the Gene Expression Omnibus (GEO, http://www.ncbi.nlm.nih.gov/geo, accessed on 25 December 2025) at the National Center for Biotechnology Information. Five high-quality synovial datasets for OA were selected: GSE206848, GSE55457, GSE143514, GSE55235, and GSE82107. Samples of rheumatoid arthritis origin that were present in some of these datasets were excluded, and only OA samples were retained. In addition, samples flagged for skeletal muscle contamination or other quality control issues were removed following the methods of Newton et al. [47]. The three datasets, GSE206848, GSE55457 and GSE143514, were combined to form the training set for model development and core gene screening, while GSE55235 and GSE82107 served as external validation cohorts to evaluate the robustness of the diagnostic genes and the predictive model. Detailed sample information can be found in Supplementary Table S2.

### 4.3. Differential Expression Analysis

First, the training dataset was merged and batch corrected. The ComBat algorithm, which is based on the empirical Bayesian parameter framework [48], was used to eliminate systematic technical differences between different platforms. Differentially expressed genes (DEGs) were identified on the basis of thresholds of an FDR-adjusted *p* value less than 0.05 and an absolute log2 fold change (|log2FC|) ≥ 0.585. To evaluate the functional correlation of these DEGs, comprehensive GO and KEGG enrichment analyses [49,50] were conducted. These analyses systematically mapped the DEGs to three ontological categories (biological process, cellular component, and molecular function) and revealed their involvement in key signaling pathways and molecular regulatory pathways.

### 4.4. Weighted Gene Coexpression Network Analysis (WGCNA)

Prior to network construction, the expression matrix was subjected to quality control and gene filtering [51]. Nonvariable genes were removed, and expression variability was quantified via the median absolute deviation (MAD). The optimal soft threshold parameter (β) was determined by evaluating the scale-free topology fit index R^2^ and mean network connectivity to ensure conformity with scale-free network properties. Subsequently, based on the TOM dissimilarity metric, hierarchical clustering was performed using the average link method, and gene clusters were identified via the dynamic tree cut algorithm. The module eigengenes (MEs) of each module were calculated, and further hierarchical clustering was conducted to analyse the independence between modules. Pearson correlation analysis was used to evaluate the association between each ME and OA status, and the module showing the strongest phenotypic correlation was defined as the core disease-associated module [48].

### 4.5. Identification of Diagnostic Feature Genes via Machine Learning

Genes overlapping between the key WGCNA module and the DEGs were adopted as candidate features for model construction. A total of 119 distinct model configurations were generated through combinations of algorithms and parameters. The feature importance scores and regression coefficients were extracted from all the models with good performance. The genes that consistently demonstrated high contribution weights in multiple high-scoring models were selected as the core diagnostic group for subsequent validation and mechanism studies.

### 4.6. Molecular Subtyping of OA and Immune Infiltration Analysis

The unsupervised consensus clustering was performed using the ConsensusClusterPlus R package based on the K-means algorithm [52]. The biological validity of the clustering results was confirmed via principal component analysis. To characterize immune microenvironment heterogeneity across subtypes, the ssGSEA algorithm was used to compute immune gene set enrichment scores for each sample. We then compared the enrichment levels of immune cell subsets and immune function pathways across subtypes and extracted the expression profiles of immune checkpoint-related genes. Collectively, these analyses systematically characterized the immune microenvironment features of distinct OA molecular subtypes.

### 4.7. Data Sources for MR Analysis

The datasets used for the mediation MR analyses were obtained from three independent public resources. The summary statistics of the cis-expression quantitative trait loci (cis-eQTLs) were downloaded from the eQTLGen consortium (https://eqtlgen.org/, accessed on 12 January 2026), which reports the associations between genetic variations and gene expression on the basis of peripheral blood samples from individuals of European ancestry. Genome-wide association study (GWAS) data for plasma metabolites were sourced from an open-access human plasma metabolome GWAS database containing summary statistics for genetic associations of 1400 circulating metabolites. The GWAS data for osteoarthritis were obtained from the OpenGWAS platform (https://opengwas.io/datasets/, accessed on 18 January 2026). The primary outcome dataset (ebi-a-GCST005812) included 32,970 individuals of European ancestry (6586 hospital-diagnosed OA cases and 26,384 controls) with complete GWAS summary statistics. For phenotypic sensitivity analyses, two additional OA GWAS datasets from the same study with distinct phenotypic definitions were also used: ebi-a-GCST005813 (4462 cases, 17,885 controls) and ebi-a-GCST005814 (10,083 cases, 40,425 controls).

### 4.8. MR Analysis

The cis-eQTL variants were used as genetic tools for measuring gene expression levels. To ensure the independence of each variant, we conducted a linkage disequilibrium (LD) clustering analysis, setting the threshold at r^2^ < 0.1 and the physical distance window at 500 kb. For each retained single-nucleotide polymorphism (SNP), we calculated the proportion of variance explained (R^2^) in gene expression and the corresponding F statistic; tools with F values lower than 10 were excluded to reduce potential weak tool bias. The inverse-variance weighted (IVW) method under a random-effects model was used as the primary approach for causal inference [53], and four complementary methods—MR–Egger regression, weighted median, simple mode, and weighted mode—were applied to verify the robustness of the primary findings [54].

### 4.9. Mediation MR Analysis

In the first step, cis-eQTL instruments for gene expression were used to perform MR for each plasma metabolite. For continuous metabolite outcomes, effect estimates are presented as odds ratios (ORs) derived from exponentiated beta coefficients, thereby ensuring graphical consistency across all MR analyses. In the second step, metabolites passing quality control filtering were taken forward as exposures, and their causal effects on OA were tested using the same analytical pipeline as the primary analysis. The proportion mediated was quantified as a percentage. The magnitude of the indirect effect was estimated via the product-of-coefficients method, calculated as the coefficient for the gene-to-metabolite pathway (β_XZ) multiplied by the coefficient for the metabolite-to-OA pathway (β_ZY). The direct effect was derived by subtracting the indirect effect from the total effect coefficient (β_XY) [55].

### 4.10. Single-Nucleus RNA-Seq Data Analysis

Single-nucleus RNA-seq data (GSE216651) were processed via Cell Ranger and Seurat (v5.0). Following SCTransform normalization, batch effects were corrected via Harmony, and cell types were annotated according to canonical marker genes. The overall change in TNFRSF10B expression was decomposed into an expression effect (change in weighted mean expression on the basis of cell proportions in the healthy group) and a composition effect (change in weighted cell proportions on the basis of the mean expression in the healthy group). CellChat was used to infer ligand–receptor interactions, and differences in interaction strength across conditions were visualized via heatmaps and chord diagrams.

### 4.11. Real-Time RT-PCR and Western Blotting

Total RNA was extracted using a column-based total RNA extraction kit (BSC52, Bioer, Hangzhou, China) according to the manufacturer’s instructions. Reverse transcription was performed using 12 μL of total RNA mixed with 3 μL of gDNA digester Mix and 5 μL of Hifair III SuperMix plus (11141ES, Yeasen, Shanghai, China) to a final volume of 20 μL. The reaction was performed according to the manufacturer’s protocol: 25 °C for 5 min, 55 °C for 15 min, and 85 °C for 5 min. The sequences of primers used in this study were as follows: for human *TNFRSF10B*, forward primer: CTGTTCTCTCTCAGGCATCATC, reverse primer: GGAAGGACTTTCTTCCACAGTAA; for human GAPDH, forward primer: CAGGAGGCATTGCTGATGAT, reverse primer: GAAGGCTGGGGCTCATTT. *GAPDH* was used as an endogenous reference gene for normalization of the RNA input. The relative gene expression level was calculated via the 2^−ΔΔCt^ method.

Total protein was extracted using RIPA lysis buffer (P0013, Beyotime, Shanghai, China) supplemented with protease inhibitors (P1045, Beyotime, Shanghai, China), followed by tissue grinding and ultrasonic lysis on ice and centrifugation at 13,400g for 15 min at 4 °C. The supernatant was collected as total protein, and protein concentration was determined via BCA assay. Immunoblotting was performed using an anti-TNFRSF10B antibody (ab8416, Abcam, Cambridge, UK) at a concentration of 2 µg/mL, an anti-β-actin antibody (20536-1-AP; Proteintech, Wuhan, China) at a dilution of 1:5000, and an HRP-conjugated secondary antibody at a dilution of 1:5000. The bound antibodies were detected via enhanced chemiluminescence (ECL) (BL520B, Biosharp, Hefei, China), and the protein levels were quantified via grayscale scanning. Normalization was carried out using β-actin (20536-1-AP; Proteintech, Wuhan, China) as the standard.

### 4.12. Immunohistochemical Staining

The TNFRSF10B protein (ab8416; Abcam, Cambridge, UK) in OA and control synovial tissues was detected via immunohistochemistry. After antigen retrieval, endogenous peroxidase activity was terminated with 3% H_2_O_2_, and non-specific binding was blocked with goat serum (AR1009, Boster, Wuhan, China). The sections were incubated with anti-TNFRSF10B primary antibody at a concentration of 5 µg/mL overnight at 4 °C, followed by incubation with HRP-conjugated secondary antibody. After staining with DAB (ZLI-9018, ZSBIO, Beijing, China) and counterstaining with hematoxylin (G1004, Servicebio, Wuhan, China), three randomly selected high-power fields (×20 magnification) per section were captured. The proportion of TNFRSF10B-positive cells was quantified using ImageJ software (version 1.54; NIH, Bethesda, MD, USA), and the average value per section was used for statistical analysis.

### 4.13. Quantification and Statistical Analysis

Statistical analysis was performed via GraphPad Prism software (version 10.0; San Diego, CA, USA). For two-group comparisons, a two-tailed Student’s *t* test was used for normally distributed variables, and the Mann–Whitney U test was applied for nonnormally distributed variables. Comparisons across multiple groups were performed via one-way analysis of variance (ANOVA).

## 5. Conclusions

Through integrative multiomics bioinformatics, Mendelian randomization-based causal inference, and clinical validation, we identified TNFRSF10B as a promising diagnostic biomarker and a candidate protective factor for OA. Furthermore, our two-step mediation MR analysis suggests that the protective effect of TNFRSF10B may be partially mediated by the alpha-tocopherol-to-sulfate ratio. Nevertheless, the underlying molecular mechanisms remain to be experimentally validated.

## Data Availability

Transcriptomic datasets are available from NCBI GEO (http://www.ncbi.nlm.nih.gov/geo). MR summary statistics were downloaded from eQTLGen (https://eqtlgen.org/) and OpenGWAS (https://opengwas.io/datasets/). Additional raw data underlying the study findings will be provided by the corresponding authors upon submission of a reasonable request.

## Supplementary Materials

The following supporting information can be downloaded at: https://www.mdpi.com/article/10.3390/ijms27188382/s1.

## Abbreviations

The following abbreviations are used in this manuscript:

AUC	Area under the curve
cis-eQTL	Cis-expression quantitative trait locus
DEGs	Differentially expressed genes
GEO	Gene Expression Omnibus
GO	Gene Ontology
GWAS	Genome-wide association study
KEGG	Kyoto Encyclopedia of Genes and Genomes
LD	Linkage disequilibrium
MAD	Median absolute deviation
MEs	Module eigengenes
MR	Mendelian randomization
OA	Osteoarthritis
ROC	Receiver operating characteristic
SNP	Single-nucleotide polymorphism
snRNA-seq	Single-nucleus RNA sequencing
TOM	Topological overlap matrix
WGCNA	Weighted gene coexpression network analysis
