# Supplementary material for: TNFRSF10B Implicated in Osteoarthritis Protection via the Alpha-Tocopherol-to-Sulfate Ratio: A Multiomics and Mendelian Randomization Study
Source: Int J Mol Sci. 2026 Sep 20;27(18):8382. doi: 10.3390/ijms27188382 (PMC13607894; doi:10.3390/ijms27188382)
Supplement: Supplementary file 1 [file ijms-27-08382-s001.zip › Supplementary Figures.pdf]

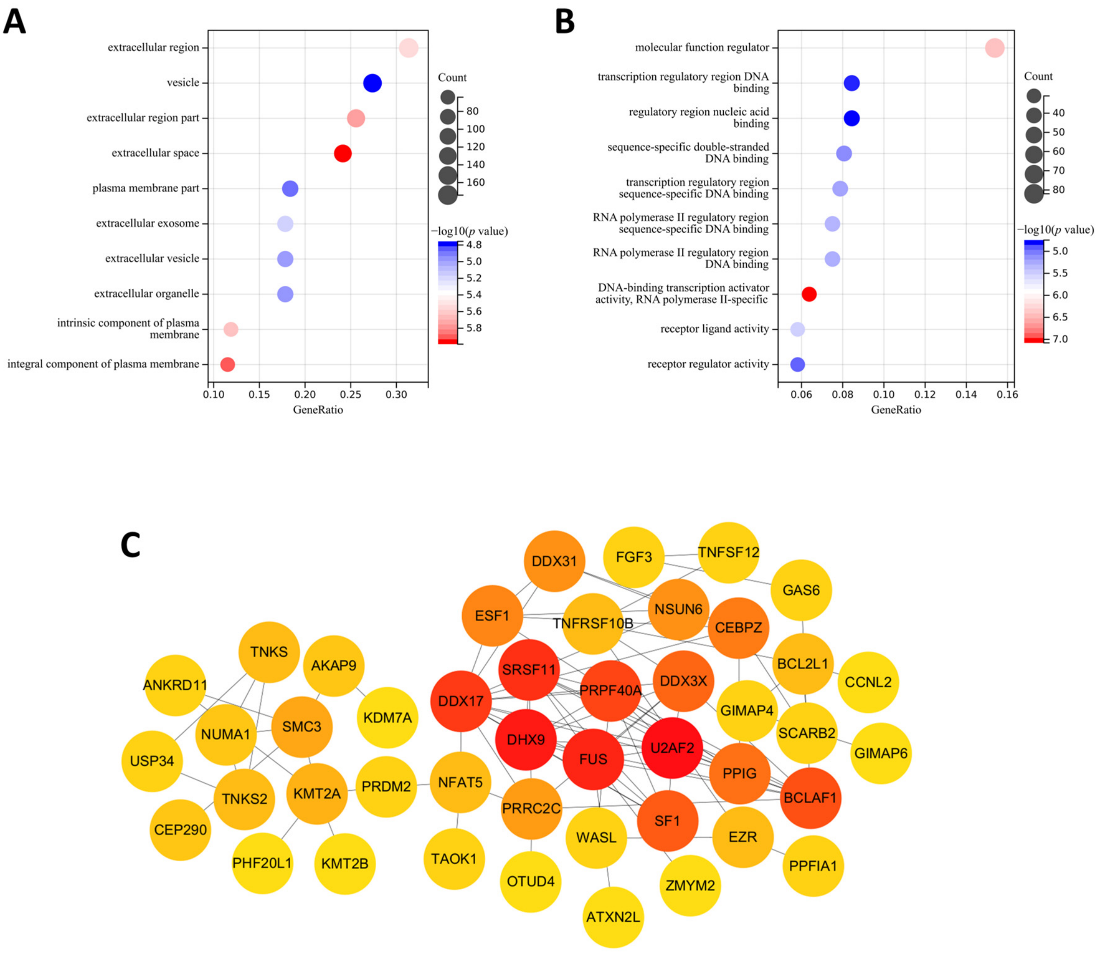


**Supplementary Figure S1.** Functional enrichment and protein interaction network of OA-related DEGs. (A) Bubble chart displaying significantly enriched Gene Ontology terms belonging to the CC category. (B) Bubble chart presenting enriched Gene Ontology functional terms under the molecular function classification. (C) PPI network of candidate core genes, with node color indicating topological importance ranked by cytoHubba.

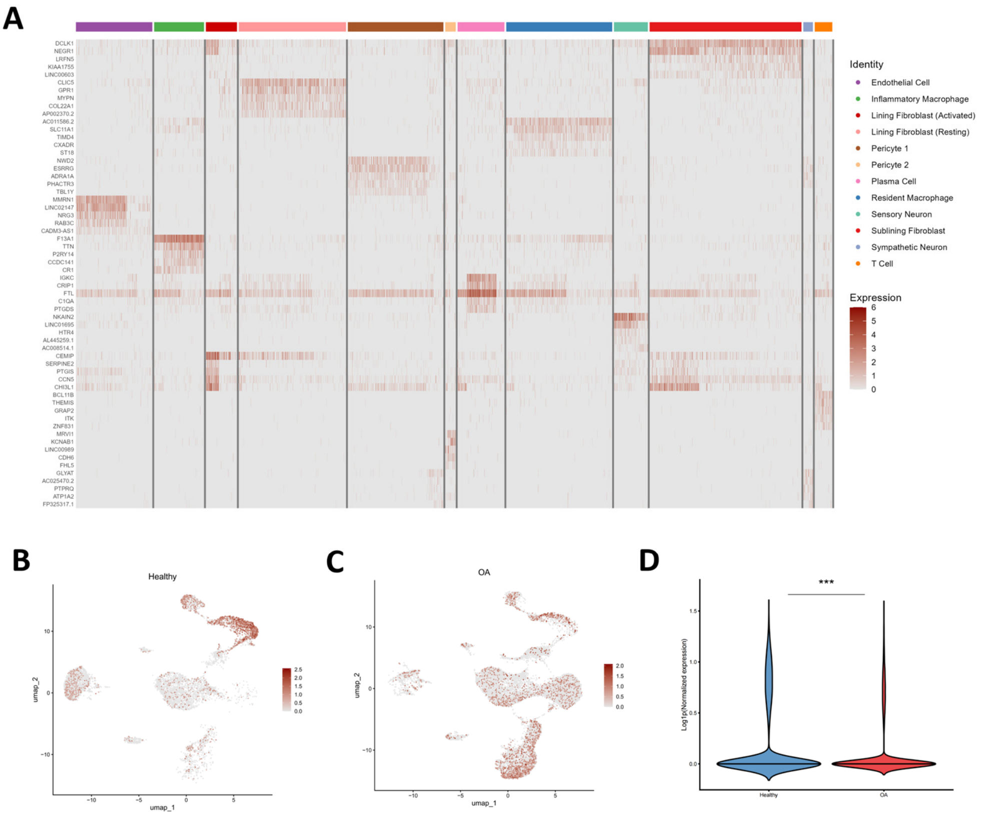


**Supplementary Figure S2.** Cell type annotation validation and intergroup comparison of TNFRSF10B expression. (A) Heatmap showing the expression of the top 5 marker genes for each cell cluster across all single nuclei, grouped by annotated cell types. (B, C) UMAP projections of TNFRSF10B expression separately for healthy and OA samples. (D) Violin plot comparing the overall log-normalized expression level of TNFRSF10B between healthy and OA synovium. Statistical significance was assessed by the Wilcoxon rank-sum test (****p* < 0.001).

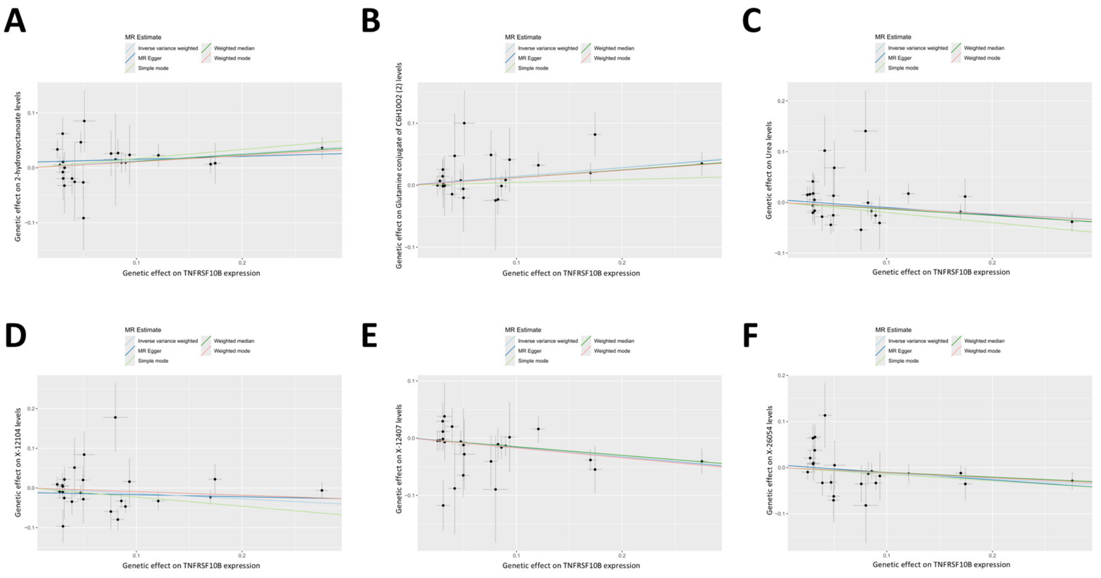


**Supplementary Figure S3.** Two-sample MR scatter plots for TNFRSF10B expression and six candidate mediator metabolites. Each panel plots per-SNP genetic effect estimates for TNFRSF10B expression (x-axis) against the corresponding metabolite level (y-axis); fitted regression lines represent causal effect estimates derived from multiple MR analytical methods. (A) 2-Hydroxyoctanoate. (B) Glutamine conjugate of C6H10O2 (2). (C) Urea. (D) X-12104. (E) X-12407. (F) X-26054.
