# Supplementary material for: TNFRSF10B Implicated in Osteoarthritis Protection via the Alpha-Tocopherol-to-Sulfate Ratio: A Multiomics and Mendelian Randomization Study
Source: Int J Mol Sci. 2026 Sep 20;27(18):8382. doi: 10.3390/ijms27188382 (PMC13607894; doi:10.3390/ijms27188382)
Supplement: Supplementary file 1 [file ijms-27-08382-s001.zip › Supplementary Tables.pdf]

**Supplementary Table S1. Clinical characteristics of the synovial tissue samples used for validation**

| Characteristic | Control group (n=5) | OA group (n=5) | *p* value |
|---|---|---|---|
| Sex, n (%) | | | 1.000 |
| Female | 2 (40.0) | 2 (40.0) | |
| Male | 3 (60.0) | 3 (60.0) | |
| Age (years), mean ± SD | 57.6 ± 5.5 | 59.4 ± 5.9 | 0.632 |
| Kellgren-Lawrence grade, n (%) | | | – |
| I-II | 5 (100.0) | 0 (0.0) | |
| III-IV | 0 (0.0) | 5 (100.0) | |

Footnotes: Data are presented as n (%) for categorical variables and mean ± SD for continuous variables. *p* values were calculated using Fisher's exact test for sex and two-tailed independent Student's t-test for age. No significant differences were found between groups regarding baseline characteristics ($p > 0.05$). Control synovial tissues were obtained from patients undergoing surgery for traumatic tibial plateau fracture, with no history of degenerative or inflammatory joint disease. OA patients were diagnosed with primary knee osteoarthritis and scheduled for total knee arthroplasty. Kellgren-Lawrence grade was used as a criterion for group allocation; therefore, no statistical comparison was performed for this variable.

**Supplementary Table S2. Basic information of datasets**

| GSE series | Type | Sample size | | Platform |
|---|---|---|---|---|
| | | Control | OA | |
| GSE206848 | mRNA | 7 | 7 | GPL570 |
| GSE55457 | mRNA | 10 | 10 | GPL96 |
| GSE143514 | mRNA | 3 | 5 | GPL20795 |
| GSE55235 | mRNA | 10 | 10 | GPL96 |
| GSE82107 | mRNA | 7 | 10 | GPL570 |

Footnotes: GSE series represent the accession numbers of datasets from the Gene Expression Omnibus (GEO) database. GPL represents the platform accession number for the microarray or sequencing platform used in each dataset. After quality control (removing two OA samples flagged for skeletal muscle contamination, as described in Methods 4.2), the final training set comprised 20 controls and 20 OA samples.

**Supplementary Table S3. Top 50 proteins in the PPI network ranked by MCC score**

| Rank | Name | Score |
|---|---|---|
| 1 | U2AF2 | 222 |
| 2 | DHX9 | 192 |
| 3 | FUS | 123 |
| 4 | SRSF11 | 122 |
| 5 | DDX17 | 112 |
| 6 | PRPF40A | 103 |
| 7 | BCLAF1 | 101 |
| 8 | SF1 | 97 |

| | | |
|---|---|---|
| 9 | DDX3X | 53 |
| 10 | PPIG | 36 |
| 11 | CEBPZ | 31 |
| 12 | ESF1 | 30 |
| 13 | DDX31 | 24 |
| 13 | NSUN6 | 24 |
| 15 | PRRC2C | 9 |
| 16 | SMC3 | 7 |
| 17 | KMT2A | 5 |
| 18 | BCL2L1 | 4 |
| 18 | TNFRSF10B | 4 |
| 18 | NFAT5 | 4 |
| 18 | TNKS | 4 |
| 18 | TNKS2 | 4 |
| 18 | EZR | 4 |
| 24 | AKAP9 | 3 |
| 24 | NUMA1 | 3 |
| 24 | CEP290 | 3 |
| 27 | FGF3 | 2 |
| 27 | TNFSF12 | 2 |
| 27 | GAS6 | 2 |
| 27 | GIMAP4 | 2 |
| 27 | RORA | 2 |
| 27 | USP34 | 2 |
| 27 | PRDM2 | 2 |
| 27 | TAOK1 | 2 |
| 27 | SCARB2 | 2 |
| 27 | ANKRD11 | 2 |
| 27 | WASL | 2 |
| 27 | PPFIA1 | 2 |
| 39 | COL6A1 | 1 |
| 39 | FBLN5 | 1 |
| 39 | ATXN2L | 1 |
| 39 | COLQ | 1 |
| 39 | PURA | 1 |
| 39 | SMC5 | 1 |
| 39 | ZMYM2 | 1 |
| 39 | CCNL2 | 1 |
| 39 | TEAD1 | 1 |
| 39 | GABPA | 1 |
| 39 | GIMAP6 | 1 |
| 39 | ICOS | 1 |

Footnotes: MCC, Maximal Clique Centrality. Scores were calculated using the cytoHubba plugin in

Cytoscape to rank proteins in the PPI network.

**Supplementary Table S4. Results of heterogeneity and pleiotropy analyses**

| Outcome | Exposure | Method | *Q* | *Q*_df | *Q*_*p*val |
|---|---|---|---|---|---|
| ebi-a-GCST005812 | TNFRSF10B | MR Egger | 21.742 | 25 | 0.651 |
| ebi-a-GCST005812 | TNFRSF10B | Inverse variance weighted | 21.805 | 26 | 0.699 |
| ebi-a-GCST005813 | TNFRSF10B | MR Egger | 19.779 | 25 | 0.758 |
| ebi-a-GCST005813 | TNFRSF10B | Inverse variance weighted | 20.678 | 26 | 0.758 |
| ebi-a-GCST005814 | TNFRSF10B | MR Egger | 21.812 | 25 | 0.647 |
| ebi-a-GCST005814 | TNFRSF10B | Inverse variance weighted | 22.168 | 26 | 0.679 |

| Outcome | Exposure | Egger_intercept | SE | *p*val |
|---|---|---|---|---|
| ebi-a-GCST005812 | TNFRSF10B | 0.002 | 0.009 | 0.805 |
| ebi-a-GCST005813 | TNFRSF10B | −0.010 | 0.011 | 0.352 |
| ebi-a-GCST005814 | TNFRSF10B | 0.004 | 0.007 | 0.556 |

Footnotes: *Q*_*p*val > 0.05 for all analyses, indicating no significant heterogeneity among instrumental variables. Egger intercept $p > 0.05$ for all analyses, indicating no evidence of directional pleiotropy. *Q*_df, degrees of freedom; SE, standard error.

**Supplementary Table S5. Mendelian randomization analysis of the ebi-a-GCST005813 and ebi-a-GCST005814 datasets**

| Outcome | Exposure | Method | nsnp | *p*val | OR | OR_lci95 | OR_uci95 |
|---|---|---|---|---|---|---|---|
| ebi-a-GCST005813 | TNFRSF10B | MR Egger | 27 | 0.160 | 0.856 | 0.694 | 1.056 |
| ebi-a-GCST005813 | TNFRSF10B | Weighted median | 27 | 0.162 | 0.877 | 0.729 | 1.054 |
| ebi-a-GCST005813 | TNFRSF10B | Inverse variance weighted | 27 | 0.001 | 0.792 | 0.692 | 0.906 |
| ebi-a-GCST005813 | TNFRSF10B | Simple mode | 27 | 0.039 | 0.704 | 0.513 | 0.966 |
| ebi-a-GCST005813 | TNFRSF10B | Weighted mode | 27 | 0.014 | 0.812 | 0.696 | 0.947 |
| ebi-a-GCST005814 | TNFRSF10B | MR Egger | 27 | 0.085 | 0.880 | 0.766 | 1.012 |
| ebi-a-GCST005814 | TNFRSF10B | Weighted median | 27 | 0.255 | 0.933 | 0.827 | 1.052 |
| ebi-a-GCST005814 | TNFRSF10B | Inverse variance weighted | 27 | 0.037 | 0.910 | 0.832 | 0.994 |
| ebi-a-GCST005814 | TNFRSF10B | Simple mode | 27 | 0.228 | 0.881 | 0.721 | 1.077 |

| | | | | | | | |
|---|---|---|---|---|---|---|---|
| ebi-a-GCST005814 | TNFRSF10B | Weighted mode | 27 | 0.060 | 0.910 | 0.828 | 1.000 |

**Footnotes:** MR, Mendelian randomization; nsnp, number of single nucleotide polymorphisms used as instrumental variables; OR, odds ratio; OR_lci95, lower bound of the 95% confidence interval for OR; OR_uci95, upper bound of the 95% confidence interval for OR. The inverse variance weighted method was used as the primary analysis. $p < 0.05$ was considered statistically significant. All ORs are per one standard deviation increase in TNFRSF10B expression.

**Supplementary Table S6. Mediation Mendelian randomization analysis of the TNFRSF10B–alpha-tocopherol-to-sulfate ratio–osteoarthritis axis**

| **Mediator** | **Indirect_Beta** | **Indirect_*p*val** | **Direct_Beta** | **Total_Beta** | **Total_*p*val** | **Mediation_Proportion** | **Mediation_Prop_(95% CI)** |
|---|---|---|---|---|---|---|---|
| Alpha-tocopherol-to-sulfate-ratio | −0.0341 | 0.017 | −0.1681 | −0.2022 | <0.001 | 16.85% | 16.85% (2.96%, 30.74%) |

**Footnotes:** MR, Mendelian randomization; CI, confidence interval; Beta, regression coefficient. The indirect, direct, and total effects were estimated using a two-step mediation MR framework. $p < 0.05$ was considered statistically significant. The mediation proportion was 16.85% (95% CI: 2.96% to 30.74%).
